# Implications of COVID‐19 for HIV Research: data sources, indicators and longitudinal analyses

**DOI:** 10.1002/jia2.25627

**Published:** 2020-10-12

**Authors:** Peter F Rebeiro, Stephany N Duda, Kara K Wools‐Kaloustian, Denis Nash, Keri N Althoff, the International epidemiology Databases to Evaluate AIDS (IeDEA)

**Affiliations:** ^1^ Department of Medicine (Divisions of Infectious Diseases & Epidemiology) & Department of Biostatistics Vanderbilt University School of Medicine Nashville TN USA; ^2^ Department of Biomedical Informatics Vanderbilt University School of Medicine Nashville TN USA; ^3^ Division of Infectious Diseases Department of Medicine Indiana University School of Medicine Indianapolis IN USA; ^4^ Institute for Implementation Science in Population Health City University of New York New York NY USA; ^5^ Department of Epidemiology Johns Hopkins Bloomberg School of Public Health Baltimore MD USA

**Keywords:** HIV care continuum, HIV epidemiology, COVID‐19, research design, data sources, longitudinal studies

## 1 INTRODUCTION

Observational research is critical to inform guidelines, policy, and the practice of HIV service delivery [1]. The COVID‐19 pandemic has profoundly affected healthcare systems and health behaviours world‐wide, including at clinics and research sites that undergird global observational HIV research [2]. We consider the impact of the COVID‐19 pandemic on the capture of relevant HIV data, indicator fidelity and analytic approaches when investigating effects of COVID‐19 itself or accounting for COVID‐related changes in service delivery and care‐seeking.

### 1.1 Data sources

Observational HIV research relies on robust data sources that accurately reflect the delivery of routine patient care, which is the underlying data‐generating mechanism. Due to the COVID‐19 pandemic, patient health behaviours and HIV clinical care models have changed. Patients may be unable to attend clinic due to COVID‐19 health concerns, reduced transportation and stay‐at‐home orders. Clinics globally have responded with increased remote interactions through telehealth, electronic patient portals, social media platforms and text and email messaging, as well as decentralized antiretroviral therapy (ART) delivery.

However, new types of care and medication delivery may not be recorded consistently in paper or electronic health record systems and many providers working off‐site may not have access to those systems for documentation [2, 3]. Even if these interactions are recorded, data may be inaccessible to researchers if stored in new systems or data fields [4]. Linked data sources such as pharmacy systems may also experience changes in data quality and content, as prescriptions are dispensed in batches for community delivery or transferred to pharmacies offering reduced‐contact dispensing [2, 3]. Mortality and other registries may experience data entry delays due to reporting delays and reduced staffing. These pandemic‐related changes are likely to be as heterogeneous across the globe as the pandemic itself, disrupting the data sources researchers have used to assess trends in key HIV‐related outcomes, resulting in unreliable and invalid measures of care [2].

### 1.2 Indicators & measurement

The HIV care continuum has become the preferred framework for understanding individual movement through various stages of HIV care, from testing and linkage to care, to retention in care, ART receipt, and ultimately, viral suppression. Barriers at various stages of the continuum have been conceptualized as “leaks,” with gaps, delays and transitions out of care seen as undesirable events that should be mitigated through intervention [5, 6].

HIV testing, diagnosis and linkage to care have been delayed due to suspension or limitation of testing programs during mandatory public quarantine or social distancing measures. Outpatient clinic visits have been shifted to remote encounters when possible, and non‐urgent care has been postponed [2, 7]. Individuals that appear to be lost to follow‐up at their usual site of care may be seeking care elsewhere and medical records may not travel with them. Delays in ART initiation and refills have occurred due to loss of insurance, limited pharmacy dispensing capabilities, and/or limited outpatient activities. CD4 and viral load monitoring – central to HIV care – may also be delayed due to less available phlebotomy services or co‐opting of equipment for COVID‐related testing [3].

Both improved data capture and alteration of care continuum metrics may therefore be required to assess the extent of care/service disruptions and reduce measurement error and misclassification [8]. More sensitive definitions of engagement and retention accounting for non‐traditional interactions may also be warranted. Many current retention metrics require clinical interactions every three to six months, but individuals successfully managed on ART and virally suppressed may need less frequent visits [6, 9]. A conditional retention measure, based on ART receipt and viral suppression prior to clinic service disruption or upon return to clinic, may therefore be more informative about the care continuum than attended or even missed visit counts alone. For example we could redefine retention status such that an individual would be successfully retained if they were virally suppressed and receiving ART both before local social isolation measures were imposed and after return to the clinic. Such a measure would be a more meaningful indication of the current state of a patient's engagement in the HIV care continuum, even if their recorded HIV care visits were not frequent enough to meet current retention definitions.

### 1.3 Analytic considerations

In addition to changes in data collection and measurement during study design and conduct, we will also need to use analytic approaches that address the potential for artifactual temporal changes in HIV indicators due to COVID‐19, selection biases and measurement errors in the data‐generating mechanisms of the care continuum. If patients attending telehealth visits are not representative of the entire cohort, if outcomes are unreliably ascertained among those lost to care, if certain measures are self‐reported remotely instead of being collected on‐site, or if discontinuities such as disruptions in care persist, appropriate epidemiologic and biostatistical methods such as inverse probability weighting, multiple imputation, double‐sampling and regression calibration should be considered [10, 11, 12]. To facilitate longer‐term trend assessments which span the COVID‐19 pandemic, analyses should also accommodate maximum flexibility, for example through the use of restricted cubic splines, piecewise regression or parametric mixture models [13, 14]. We must continue to assess the local clinical context to obtain more information relevant to HIV care changes induced by the COVID‐19 pandemic and inform these approaches [15].

## 2 CONCLUSION

Future HIV‐related studies and public health goals require a new COVID‐19‐informed paradigm for the collection and use of observational cohort data. HIV cohorts must capture pandemic‐driven changes in data sources, clinic activities and local policies to inform analyses. Our ability to leverage epidemiologic evidence to inform clinical, programmatic and public health practice is only as strong as the inferences derived from these analyses are valid and robust to the challenges in HIV care and research that we now face due to the pandemic. Healthcare organizations and public health agencies should revise HIV care continuum measures and analytic strategies. Funding for such work is critical, even in times of economic crisis, so that COVID‐19 does not derail the global fight to End the HIV Epidemic.

## COMPETING INTERESTS

The authors declare no competing interests.

## AUTHORS’ CONTRIBUTIONS

PFR, SND and KNA developed the idea and wrote the initial draft. KKW and DN revised the manuscript and provided expert input. All authors approved the final manuscript.

## FUNDING

The International Epidemiology Databases to Evaluate AIDS (IeDEA) is supported by the U.S. National Institutes of Health’s National Institute of Allergy and Infectious Diseases (NIAID), the *Eunice Kennedy Shriver* National Institute of Child Health and Human Development, the National Cancer Institute, the National Institute of Mental Health, the National Institute on Drug Abuse, the National Heart, Lung, and Blood Institute, the National Institute on Alcohol Abuse and Alcoholism, the National Institute of Diabetes and Digestive and Kidney Diseases, the Fogarty International Center, and the National Library of Medicine: CCASAnet, U01AI069923; Central Africa, U01AI096299; East Africa, U01AI069911; NA‐ACCORD, U01AI069918. Informatics resources are supported by the Harmonist project, R24AI124872. PFR received additional funding from NIAID (K01‐AI131895, R21‐AI145686).

## 3 DISCLAIMER

This work is solely the responsibility of the authors and does not necessarily represent the official views of any of the institutions mentioned above.
